# Recent Advances in the Diagnosis, Staging, Treatment, and Prognosis of Advanced Gastric Cancer: A Literature Review

**DOI:** 10.3389/fmed.2021.744839

**Published:** 2021-10-26

**Authors:** Zhi-da Chen, Peng-fei Zhang, Hong-qing Xi, Bo Wei, Lin Chen, Yun Tang

**Affiliations:** ^1^Department of General Surgery, First Medical Center of Chinese People's Liberation Army of China (PLA) General Hospital, Beijing, China; ^2^Department of Oncology, First Medical Center of Chinese People's Liberation Army of China (PLA) General Hospital, Beijing, China

**Keywords:** advanced gastric cancer, treatment, diagnosis, staging, prognosis

## Abstract

Gastric cancer is one of the most common cause of cancer related deaths worldwide which results in malignant tumors in the digestive tract. The only radical treatment option available is surgical resection. Recently, the implementation of neoadjuvant chemotherapy resulted in 5-year survival rates of 95% for early gastric cancer. The main reason of treatment failure is that early diagnosis is minimal, with many patients presenting advanced stages. Hence, the greatest benefit of radical resection is missed. Consequently, the main therapeutic approach for advanced gastric cancer is combined surgery with neoadjuvant chemotherapy, targeted therapy, or immunotherapy. In this review, we will discuss the various treatment options for advanced gastric cancer. Clinical practice and clinical research is the most practical way of reaching new advents in terms of patients' characteristics, optimum drug choice, and better prognosis. With the recent advances in gastric cancer diagnosis, staging, treatment, and prognosis, we are evident that the improvement of survival in this patient population is just a matter of time.

## Introduction

Gastric cancer, the third leading cause of cancer-related mortality, is a prevalent gastrointestinal tumor, particularly in China, with about 400,000 new cases each year (1). According to a report published in 2020, each year over one million cases of gastric cancer are diagnosed worldwide. It is the 5th most diagnosed and the 7th most prevalent cancer in the world (2). Each year gastric cancer accounts for 783,000 deaths which makes it the third most deadly cancer among males worldwide. 8.3% of all cancer deaths are associated with gastric cancer. In developed countries, stomach cancer is 2.2 times more likely to affect males than females (3). Gastric cancer can be classified, based on staging, into two types: early stage and advanced stage. Of note, the first type of cancer is limited to the mucosa and submucosa, regardless of the size of the tumor or the presence/absence of lymph node metastasis. On the other hand, cancers that extend beyond the level of gastric submucosa and into the muscle layer are classified as middle gastric cancers. Meanwhile, the advanced-stage type is known when the tumor cells reach the level of subserosa or beyond it to infiltrate the surrounding organs. The staging of gastric cancer reflects the efficacy of the treatment plan (4).

Surgery has always been the main approach in the treatment of gastric cancer, mainly through D2 resection. D2 can be defined as dissection of all the Group 1 and Group 2 nodes. Different locations of the cancer require different forms of gastric resections within the stomach. Therefore, four levels of lymph node dissections (D1–D4) are defined, where specified lymph nodes are dissected for a given type of resection from assigned lymphatic stations. These levels were defined by the General Rules for the Gastric Cancer Study in Surgery and Pathology by the Japanese Research Society for Gastric Cancer in 1962 and were revised in 1994. Several studies reported that only D2 dissection has proven significantly beneficial to the outcomes of patients with gastric cancer (5). A standard D2 resection of gastric cancer involves the resection of a part of or the whole stomach, N1 (groups 1:6), and N2 (groups 7:11) lymph nodes, and the greater and lesser omenta. The spleen and pancreatic tail may also be resected during the D2 resection procedure in cases with proximal stomach cancer to remove groups 10 and 11 of lymph nodes (6). Even with the current advances in the surgical approaches (D2 and laparoscopic resection) in cases with gastric cancer, the outcome is still not favorable, with a 5-year survival rate of around 45% in cases with advanced gastric cancer (7, 8).

Few biomarkers attributed to gastric cancer have been translated into molecularly targeted therapies. HER-2 is one of the therapeutic gastric cancer biomarker An open-label, randomized-controlled, phase III clinical trial investigated the efficacy of trastuzumab (a monoclonal antibody) against HER-2, in combination with chemotherapy for the treatment of HER-2-positive gastric cancers, and reported that trastuzumab treatment significantly improved overall survival and disease free survival times as compared to chemotherapy alone (9).

Another biomarker found for gastric cancer immunotherapy is PD-L1. The ATTRACTION-2 study, a phase III, randomized clinical trial, compared the effectiveness of a monoclonal antibody nivolumab against PD-L1, in individuals with advanced gastric cancer and revealed that nivolumab greatly increased overall survival and reduced the risk of mortality (10).

Improving the accuracy of pre-operative diagnosis and clinical staging of gastric cancer will result in a significant impact on the prompt management approach of gastric cancer. Likewise, this will lead to the proper identification of the most appropriate treatment option for such patients. Therefore, we conducted the current investigation to review the available literature to provide helpful insight into the proper diagnosis, staging, and comprehensive treatment of cases with advanced gastric cancer.

## Progress in The Treatment Approaches of Gastric Cancer

### Surgery

The surgical approach of gastric cancer has ranged from open surgery to laparoscopic resection; the surgical treatment of gastric cancer has witnessed a significant leap in terms of outcomes. From early-stage gastric cancer to advanced gastric cancer, the indications for laparoscopic surgery has notably expanded, with the confirmation of its efficacy and safety by the growing body of available evidence (11–13).

Robotic surgery has been acknowledged as a better surgical approach compared to laparoscopic resection in terms of avoiding the drawbacks of the later, as it provides the following advantages: seven degrees of freedom, a tremor-filtering system, the ability to scale motion, and a three-dimensional (3D) vision system that significantly impacts a surgeon's dexterity, particularly upon dealing with tissues in a narrow field of vision (14). That being said, the mean operative time of robotic surgery has been reported to be longer compared to laparoscopic or open resection. Meanwhile, the only significant difference in favor of robotic surgery is the reduction in intra-operative blood loss (15). Therefore, the current focus should be directed toward the development of a new direction in laparoscopic surgery related to the saving of human resources while increasing the precision of the surgical approach.

The use of single-port (SPLG) and reduced-port laparoscopic gastrectomy (RPLG) is now becoming more mature and is currently being investigated by clinical trials. The application of the minimally-invasive approach via SPLG and RPLG would minimize associated trauma. Such advances in single-site surgeries have enabled surgeons to perform RPLG and SPLG via the robotic approach, and therefore, eliminating the restrictions on the movement of the surgical instrument. In a single-arm, phase I/II clinical trial investigating the efficacy and safety of conducting RPLG by a single surgeon, it was found that among 19 patients who underwent RPLG, none of them required intra-operative conversion to laparoscopic or open surgery nor had major complications during RPLG surgery (16). Therefore, it is suggested that RPLG offers a safe and effective alternative in managing cases with early-stage gastric cancer. It is also suggested that this approach could be applied in high-advanced cases as well (17). SPLG is the reduced port technique on account of surgical approaches because the operation is performed through a single incision in the abdominal wall. It is an extremely minimally invasive method, theoretically providing less post-operative pain, improved cosmetic results and earlier recovery after surgery compared to conventional multiport laparoscopic gastrectomy. SPLG is thought to be an optimal method to promote and maximize patient's quality of life in the acute post-operative phase (18, 19).

As a country with a relatively high incidence rate of gastric cancer, experts in minimally-invasive gastrointestinal surgery in China established the “Chinese Laparoscopic Gastrointestinal Surgery Study (CLASS) trial” in 2009, with the primary aim to improve the lives of patients through clinical research on the outcomes of laparoscopic surgery. In 2019, the Chinese Laparoscopic Gastrointestinal Surgery Study (CLASS) Group published the results of their CLASS-01 trial. The CLASS-01 is an open-label, randomized clinical trial, which was conducted at the level of 14 centers throughout China (20). A total of 1,056 patients with stages T2, T3, or T4a gastric cancer without distant metastases were studied during the period from September 2012 to December 2014. Recruited patients were allocated to receive either laparoscopic distal gastric resection (528 cases) or open distal gastric resection with D2 lymphadenectomy (528 cases). The authors noted that the 3-year disease-free survival (DFS) rate did not significantly change between groups: 76.5% in the laparoscopic arm and 77.8% in the open surgery arm. Furthermore, the 3-year OS rate in the laparoscopic group did not significantly differ from that in the open surgery arm (83.1 vs. 85.2%, *P* = 0.28). Therefore, it was stated that the laparoscopic approach in patients, with pre-operative clinical staging of a locally advanced gastric cancer, did not lead to an inferior DFS outcome at 3 years.

Upon reviewing the recent advances in the laparoscopic resection in gastric cancer, we have noted that the continuous enhancement of this minimally invasive procedure, along with the saving of human resources, will become the future trend in managing patients with gastric cancer. At the same time, researchers have also begun to develop and implement various artificial intelligence (AI) software in laparoscopy gastrectomy. This will result in a significant impact on patients-related outcomes as well as surgeons.

### Adjuvant Chemotherapy

Adjuvant chemotherapy is recommended in completely-resected T2N0, T3, or T4 gastric adenocarcinoma, particularly in those who did not receive neoadjuvant therapy (21). The Japanese Adjuvant Chemotherapy Trial of TS-1 for Gastric Cancer (ACTS-GC) highlighted the benefits of S-1, an oral fluoropyrimidine, adjuvant therapy for 1 year (22). This trial included a total of 1,059 patients with stage II or III gastric cancer, who underwent D2 surgery, were randomly assigned to receive either S-1 (6-week cycles: 2 weeks without and 4 weeks with S-1 at a dose of 80–120 mg/m^2^/day for 1 year) or surgery alone. The study showed significantly better 3-year survival in the S1 arm compared to surgery (80.1 vs. 70.1%), respectively.

The CLASSIC trial, which was conducted in South Korea, China, and Taiwan, included 1,035 patients with stage II, IIIA, or IIIB gastric cancer (23). Patients were randomly divided into two groups after D2 resection. One group received combined therapy of oral capecitabine of eight 3-week cycles (at a dose of 1,000 mg/m^2^/twice a day on days 1–14 each cycle) and intravenous oxaliplatin (130 mg/m^2^/on day 1 of each cycle for 6 months after surgery). Meanwhile, the other group received surgery only. Patients who received the combined adjuvant chemotherapy had significantly higher 3-year disease-free survival (DFS) of 74% compared to the 59% 3-year DFS in those who underwent surgery alone. This observation highlights the fact that XELOX (capecitabine plus oxaliplatin) adjuvant chemotherapy, when administered for 6 months, can significantly reduce the risk of post-operative recurrence while improving DFS in patients with gastric cancer. The results of this study further establish the status of the XELOX regimen as a standard chemotherapeutic agent for combined therapy in the population with gastric cancers of stages II or III.

A Japanese, randomized controlled clinical trial (JACCROGC-7) was published in the American Society of Clinical Oncology (ASCO) conference in 2018 (24). Patients with stage III gastric cancer after radical gastrectomy (D2, R0) were randomly divided into two groups. The first group (experimental) received S-1 combined with Docetaxel, while the second group (control) received S-1 alone. The results of this study showed that the relapse-free survival (RFS) in the experimental group was significantly higher than that of the control group at 3 years (65.9 vs. 49.6%, *P* = 0.0007). The analysis also revealed that S-1 combined with docetaxel significantly reduced the risk of all types of recurrence, including hematogenous, lymphatic, and peritoneal. This trial highlighted the promising effects of combined S-1 and docetaxel over S-1 alone (highly significant results). This study proved the value of taxanes in the adjuvant treatment of stage III gastric cancer. Recently, combined S-1 and docetaxel regimen has become the main focus as an effective option for the treatment of patients with stage III gastric cancer following D2 surgery (24, 25).

### Intraperitoneal Hyperthermic Chemotherapy

The management approach of peritoneal metastasis from cancer of gastric-origin has improved dramatically with the use of cytoreductive surgery (CRS) and hyperthermic intraperitoneal chemotherapy (HIPEC) (26, 27). However, this is only applicable to a certain subgroup of patients. In the same context, the value of the prophylactic use or the combined therapy with HIPEC in high-risk individuals or those with positive cytology is still to be confirmed by larger and more robust randomized controlled trials.

The randomized trial of Yonemura et al. (28) showed that antitumor therapy in the form of (300 mg cisplatin plus 30 mg mitomycin) in addition to continuous hyperthermic peritoneal perfusion (CHPP), containing warmed physiological saline resulted in beneficial outcomes in patients with gastric cancer and peritoneal dissemination (41 patients). It was reported that the 50% survival period, 3- and 5-year survival rates of CHPP-treated cases were 398 days, 28.5 and 12%, respectively. Moreover, a single course of CHPP, along with the combined therapy (cisplatin and mitomycin), resulted in the disappearance of ascites in 7 of ascetic cases. In the prospective randomized study by Liang et al. (29), the intraperitoneal infusion of chemotherapy was carried out with a solution of 50 mg of mitomycin C and 375 mg of activated carbon for post-operative prophylaxis of peritoneal carcinomatosis in patients with gastric cancer. After 8 months of observation, the 3- and 5-year post-operative recurrence-free survival (RFS) rates were noted to be significantly higher in patients who were administered intraperitoneal mitomycin C plus carbon adsorbent compared to the control group, with rates of (70.16 vs. 27.09%, *P* < 0.01) and (44.51 vs. 14.45%, *P* < 0.01), respectively.

Since the application of HIPEC, researchers around the world had put tremendous efforts into improving this approach. Gastrectomy, combined with HIPEC, may lead to the prolongation of survival in patients with stage IIIB gastric cancer (30). Recently, a meta-analysis reported that HIPEC may improve the OS of patients who undergo surgical resection for advanced gastric cancer, and may also help in the prevention of local peritoneal recurrence among cases with serosal invasion in gastric cancer (31). More importantly, the results of the GASTRICHIP randomized clinical trial are expected to be published in 2025 (32). This trial is going to examining the effects of HIPEC with oxaliplatin in cases with gastric cancer that involves the serosa and/or lymph nodal involvement and/or with positive cytological analysis during peritoneal washing.

### Molecular-Targeted Therapy and Immunotherapy

**Figure 1:**
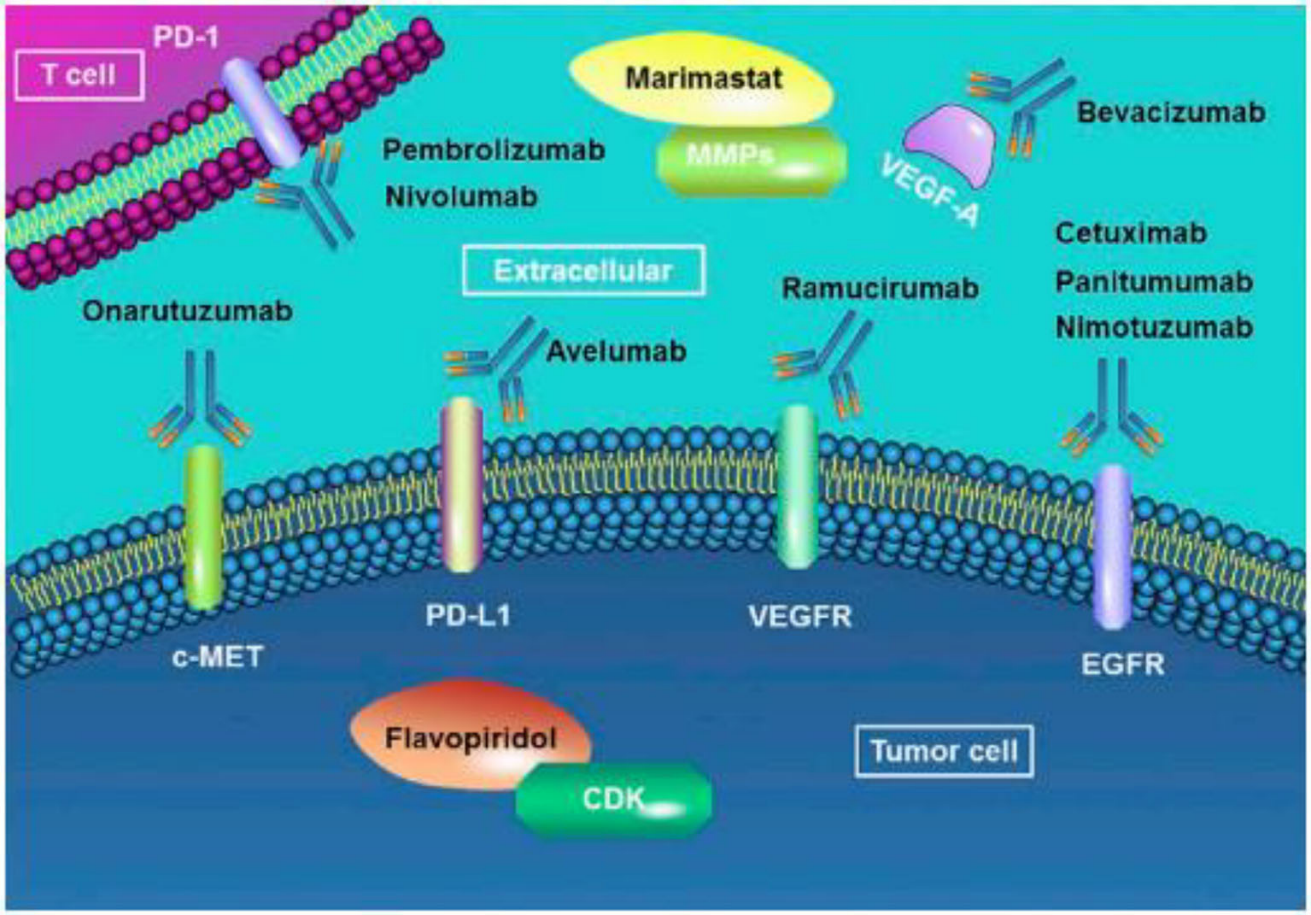
Molecular-targeted therapies of gastric cancer. EGFR, Epidermal Growth Factor Receptor; VEGFR, Vascular Endothelial Growth Factor Receptor. Adopted from the study of Song et al. (33).

In the context of precision treatment, chemotherapy combined with targeted drugs to improve efficacy has been a research hotspot and clinical focus in recent years (Figure 1). The multicenter, randomized, phase III ToGA (Trastuzumab for Gastric Cancer) trial examined the comparative efficacy chemotherapy doublet regimens in the form of (capecitabine plus cisplatin) or (5-fluorouracil plus cisplatin), which were given every 3 weeks for a total of six cycles, and chemotherapy plus intravenous trastuzumab in a group of patients with HER-2 positive gastric cancer (9). Trastuzumab plus chemotherapy was noted to result in a significantly higher median overall survival compared to the chemotherapy-alone arm (13.8 vs. 11.1 months), respectively. In patients with high HER-2 expression, trastuzumab significantly improved their median overall survival to reach 16 months compared to the 11.8 months in those assigned to chemotherapy only. Furthermore, patients who received trastuzumab in addition to chemotherapy had significantly higher median PFS compared to those who received chemotherapy alone (6.7 vs. 5.5 months), respectively.

In the setting of 2nd line treatment, ramucirumab resulted in a significant clinical improvement in previously-treated patients with gastric cancer both as stand-alone therapy and as an adjuvant to paclitaxel. The REGARD trial examined the efficacy of ramucirumab in patients with gastric adenocarcinoma (34). Patients received either intravenous ramucirumab (8 mg/kg) or placebo 2 weeks after progression following first-line chemotherapy. Ramucirumab resulted in a significantly longer median overall survival compared to placebo (5.2 vs. 3.8 months), with a 6-month PFS rate of 41.8 and 31.6%, respectively. This observation was also confirmed in the double-blinded randomized phase II RAINBOW trial, where patients with gastric adenocarcinoma received either (paclitaxel 80 mg/m^2^/days 1, 8, 15 plus ramucirumab) or placebo (days 1 and 15) (35). The combined therapy of ramucirumab and paclitaxel resulted in significantly higher OS (9.63 vs. 7.26 months), with further improvement in PFS and RR as well.

However, in the setting of 1st line treatment, ramucirumab failed to result in the same efficacy observed in 2nd-line treatment trials. In a double-blinded phase II clinical trials involving patients with advanced gastric and esophageal cancer, patients were randomized to receive either (FOLFOX chemotherapy plus ramucirumab) or (FOLFOX plus placebo) (36). Even though the combined therapy of FOLFOX and ramucirumab resulted in a significant improvement in disease control rate; however, the median PFS was insignificant to the placebo group (6.4 vs. 6.7 months, *P* = 0.89). Several ongoing clinical studies will further explore the advantages and disadvantages of anti-vascular or immune-targeted drugs combined with chemotherapy, hoping that the combined treatment can result in much better outcomes.

### Accurate Molecular Gene Therapy

In recent years, with the rapid development of cancer genomics and transcriptomics, researchers have been able to comprehensively understand the genomic characteristics, gene expression profiles, and proteomics information of gastric cancer. Moreover, they have begun to classify it as a “molecular” subtype, while, in theory, it reflects the biological behavior of gastric cancer. Although still in its preliminary phase, the new classification of gastric cancer still has the potential to promote more accurate clinical trial design, more optimal allocation of targeted therapeutic doses, and better patient outcomes.

Recently, the identification of genomic and molecular characterization of gastric cancer have shed light on the heterogeneity and potential targeted therapies for four different subtypes of gastric cancer. The Cancer Genome Atlas (TCGA) network classified gastric cancer into four subtypes: Chromosomal instability (CIN), microsatellite instable (MSI) tumors, genomically stable (GS) tumors, and Epstein-Barr virus (EBV)-positive tumors (37).

CIN-type gastric cancer accounts for about 50% of gastric cancer (38). It is characterized by mutations in the level of cytogenetics in somatic cells, which particularly involves the loci that control mitotic checkpoints. Therefore, it plays the role of a housekeeping gene in cancer formation. Altogether, CIN is associated with a set of “key genes,” such as oncogenes and tumor suppressor genes, which can act as future therapeutic targets by inhibitory agents (34). Of note, the CIN subtype is full of mutations in TP53 gene and receptor tyrosine kinases (RTKs). It is also associated with amplified cell cycle genes [i.e., Cyclin E1, Cyclin D1, and Cyclin-dependent kinase 6; (39)]. Furthermore, CIN gastric cancer subtype displays amplification in oncogene pathways, including receptor tyrosine kinase (RTK), rat sarcoma gene (RAS), and mitogen-activated protein kinase (MAPK) signaling, which includes human epidermal growth factor receptor 2 (HER2), BRAF, epidermal growth factor (EGFR), mesenchymal-epithelial transition (MET), fibroblast growth factor receptor 2 (FGFR2), and RAS (37, 40).

MSI type accounts for about 15–30% of gastric cancers; most of them are intestinal gastric cancers. They occur in the distal part of the stomach and are more common in women; they are age-related (41, 42). MSI-type gastric cancer is mainly caused by genetic changes caused by the loss of function of DNA mismatch repair system, due to the somatic or germ line epigenetic changes of mismatch repair (MMR) genes. This change may be caused by a DNA mismatch repair (MMR) dysfunction caused by a mutation between the DNA mismatch repair gene 1 (MLH1) or the mismatch repair gene 2 (MSH2) (43).

GS gastric cancer accounts for about 20%, with the same incidence in men and women (37). The main somatic alterations noted in GS gastric cancer subtype include CDH1, ARID1A, and RAS homologous gene family member A (RHOA). Moreover, a recurrent inter-chromosomal translocation between CLDN18 and ARHGAP26 involved in cell motility was noted in GS gastric cancers (37).

EBV-positive subtype accounts for about 9% of gastric cancer that is known to have variable clinicopathological characteristics (37). Recently, a meta-analysis a substantial association between EBV infection and the risk of gastric cancer; patients with EBV infection are at higher risk of having gastric cancer by a factor of 10 (95% CI: 5.89–17.29) (44). According to TCGA network reports, PD-1 is frequently amplified in EBV-positive gastric cancer, suggesting the high immunogenicity of this subtype (45). In addition to PD-1 expression, PIK3CA gene mutations, DNA hypermethylation, and Janus kinase 2 (JAK2) gene mutations are also related to EBV-positive gastric cancer (37, 38, 40–42, 44).

## Multikinase Inhibitors for The Treatment of Gastric Cancer

Multikinase inhibitors including ramucirumab (anti-VEGFR2), foretinib (anti-MET and anti-VEGFRs), and trastuzumab (anti-HER2/neu) are significantly used for the treatment of gastric cancer. In a study, Regorafenib showed dose-dependent inhibition in eight gastric cancer xenograft models with no evident toxicity or significant decreases in bodyweight. Regorafenib (10 mg/kg/day) inhibited tumor growth in all eight models resulting in reduced tumor weight. Regorafenib induced apoptosis in seven models, reduced tumor angiogenesis 3- to 11-folds and reduced tumor proliferation 2- to 5-folds in six of the eight models [all *p* < 0.05; (46)].

## Age and Diet Risk Factors Associated With Gastric Cancer Development

The important risk factors include *Helicobacter pylori* infection, obesity, smoking, age, alcohol consumption, diet, and inherited cancer syndromes. Gastric cancer can occur in younger people, but the risk increases as a person get older. The majority of people diagnosed with gastric cancer are in their 60s, 70s, or 80s *H. pylori* is a WHO classified type 1 carcinogen and its effects on gastric cancer appear multifactorial which follows a stepwise pathway toward malignancy. Studies have indicated *H. pylori* infection as an independent risk factor for gastric cancer, with a 3- to 6-fold increased risk compared to those without the infection. There is a higher risk of gastric cancer in non-Caucasian populations. In the United States, the highest incidence is found in the Asian and Native American populations. Both sex and race affect the risk of disease development and mortality rate. The highest mortality rate based upon ethnicity is of African American males which is 12.4/100,000. Furthermore, many behavioral and environmental factors affect the development of gastric carcinoma (47). Smoking is now considered an important contributor. In 1997, a meta-analysis revealed a 44% increase in risk for gastric cancer for smokers. In another meta-analysis, this increased risk was reported as 60% for men and 20% for women. Alcohol has also been identified as a risk factor for disease progression and the combined effect of alcohol and smoking increases the risk of gastric cancer 5-folds (48). Whereas, diets with high amounts of fresh fruits, vegetables, salt and processed meat have been shown to have a protective association with gastric cancer. Obese individuals have an increased risk for cardia type gastric cancer (47).

## Others Risk Factors Associated With Gastric Cancer Development

Different factors including diet and infectious agents play a significant role in gastric cancer development. An expert panel from the World Cancer Research Fund declared that high intakes of salt and salty food increase the risk of gastric cancer. A positive association with salt has been reported, as salt directly acts on the stomach lining, destroying the mucosal barrier, causing gastritis which increases epithelial proliferation. Interaction between diet and *H. pylori* infection with a high risk of gastric cancer has also been proposed. *Helicobacter pylori* infection is a strong risk factor for gastric cancer. It possesses a potent urease which allows it to survive in the acid microenvironment of the gastric lumen (49). Other factors including certain lipopolysaccharide that reduce the host immune response activity and the expression of adhesins that confer intimate adherence to the gastric epithelium also contribute to disease development. Few pieces of evidence indicate the role of Epstein-Barr virus (EBV) in the etiology of gastric cancers. Studies around the world have reported the presence of the EBV in 5–16% of gastric adenocarcinomas. A meta-analysis including 70 articles revealed that the overall EBV positivity was 8.7% among different gastric cancer cases and that EBV associated adenocarcinomas were more frequent in males than females (50).

## Different Stages of Gastric Cancer

Stages of gastric cancer can be explained with the aid of the TNM staging system which is used by doctors in cancer diagnostic. The “T” in the TNM staging system is for describing how far the tumor has grown into the stomach wall and its size is measured in centimeters (cm). The “N” is used for cancer spread to nearby lymph nodes and “M” in the TNM system describes whether cancer has spread to other parts of the body, called metastasis (51).

## Staging of Advanced Gastric Cancer

Gastric cancer staging is based on the TNM classification, according to the Union for International Cancer Control (UICC) (52)/American Joint Cancer Committee (AJCC) guidelines (53). The latest version of the tumor nodes metases (TNM) staging classification (8th edition) was released in October 2016. The staging system can effectively predict the prognosis of patients and guide clinicians to choose the optimal treatment plan. With the improvement of imaging technology, most gastric cancers can be basically diagnosed through electronic gastroscopy, gastrointestinal angiography, gastroscopy ultrasound, computed tomography (CT), magnetic resonance imaging (MRI), and positron emission tomography (PET). Post-operative routine pathological results can basically determine the overall staging; however, the current staging method still has some limitations and inaccuracies. In order to guide further treatment and prognostic evaluation after surgery, more scientific and systematic pre-operative, intra-operative, and post-operative staging methods are needed. Tables 1, 2 (54) showed, respectively, the classification of TNM staging and TNM staging of cancer.

**Table 1:** Shows classification of TNM staging system used for cancer diagnosis.

**Staging**	**TNM classification**
0	Tis, N0, M0
IA	T1, N0, M0
IB	T1, N1, M0 T2, N1, M0
IIA	T1, N2, M0 T2, N1, M0
IIB	T1, N3a, M0 T2, N2, M0 T3, N1, M0 T4a, N0, M0
IIIA	T2, N3a, M0 T3, N2, M0 T4a, N1, M0 T4b, N0, M0
IIIB	T1 or T2, N3b, M0 T3, N3a, M0 T4a, N3a, M0 T4b, N1 or N2, M0
IIIC	T3 or T4a, N3b, M0 T4b, N3a or N3b, M0
IV	Any T, Any N, M1

**Table 2:** Shows TNM staging of cancer.

T0	No evidence of primary tumor
Tis	The cancer is found only in cells on epithelium and has not spread to any other layers of the stomach.
T1	Tumbor has grown through the lining and into connective tissue
T2	Tumbor has grown into the muscle layer of the stomach
T3	Tumbor has spread through all the muscle layer and outer lining but not to organs and tissues
T4	Tumbor has grown to nearby tissues and organs
T4a	Tumbor has grown into the serosa
T4b	Tumbor has grown into organs surrounding the stomach
N0	Cancer has not spread to lymph nodes
N1	Cancer has spread to one to two regional lymph nodes
N2	Cancer has spread to three to six regional lymph nodes
N3	Cancer has spread to seven or more regional lymph nodes
N3a	Cancer has spread to seven to 15 regional lymph nodes
N3b	Cancer has spread to 16 or more regional lymph nodes
M0	No metastasis
M1	Metastasis

### Pre-operative Staging

Pre-operative staging is of great importance in guiding the choice of the most appropriate treatment option, particularly the choice of the surgical plan. At present, pre-operative auxiliary examinations, which are commonly used in clinical practice, mainly include gastroscopy ultrasound, gastrointestinal angiography, gastroscopy, CT, MRI, as well as the other aforementioned imaging modalities. In recent years, the use of new technologies such as multi-slice spiral computed tomography (MSCT), multi-detector computed tomography (MDCT), and PET-CT has greatly improved the accuracy of pre-operative staging of advanced gastric cancer (55).

Endoscopic ultrasound (EUS) is a new type of examination that combines endoscopy and ultrasound. It was the first method to determine the T stage of gastric cancer prior to surgery. The accuracy of EUS in determining the T stage of gastric cancer is about 80.3%; however, its accuracy in differentiating mucosal from submucosal cancer is about 63.6% (56). Meanwhile, a recent Cochrane meta-analysis of 50 studies revealed that the use of EUS might be more reliable in successfully identifying T3–T4 cases compared to T1–T2 cases, with sensitivity and specificity of 86 and 90%, respectively. However, the results showed considerable (non-negligible) heterogeneity and the meta-analysis could not identify the exact cause for the resulting heterogeneity. Therefore, their results should be carefully interpreted with caution till more robust meta-analysis is able to identify the reason for the reported heterogeneity (57). That being said, there are certain limitations to the N and M stages, especially the M stage, in which EUS cannot provide a conclusive diagnosis (58–60).

CT can accurately observe the depth of gastric cancer invasion, peripheral organ invasion, lymph node metastasis, and distant tumor metastasis. This is considered as the main method of pre-operative staging of advanced gastric cancer. However, it still has limitations regarding the detection of early gastric cancer and early metastatic nodules. Multi-slice spiral CT (MSCT) has the functions of 3-dimensional (3D) imaging, volume scanning, dynamic enhanced scanning, and thin layer reconstruction technology. Therefore, MSCT has significantly improved the accuracy of T and N staging. In the same context, in general, MSCT provides increased scanning speed, improved scanning resolution, and thinner slices compared to conventional CT. Furthermore, enhanced scanning provides a more accurate staging approach in cases with advanced gastric cancer before surgery, as the most appropriate treatment plan can be formulated and evaluated through the proper staging of gastric cancer before radical resection surgery. The surgical schemes have high clinical reference value. The accuracy of MDCT in detecting distant metastasis of gastric cancer is as high as 94.5%, but the sensitivity to gastric cancer peritoneal metastasis is of relatively low value. Of note, a study which is published in 2020 found that multiplanar reformations (MPR) and volume rendering (VR) of MDCT provide more accuracy than MDCT axial images in diagnosing all stages of gastric cancer [92.5 vs. 80%; *P* =0.0009; (61)].

PET-CT detects tumors and their metastatic lesions from the metabolic level based on the intake of 18F-fluorodeoxyglucose. Although the spatial resolution is low, it has high sensitivity and specificity. CT is easy to ignore micro-metastasis of the liver; however, PET scans can successfully identifying these lesions based on the high metabolic background of cancer metastases. The complementary advantages of PET and CT can significantly improve the accuracy of tumor diagnosis and staging, especially tumors with small lesions and high metabolic rate. PET-CT is worth popularizing in the pre-operative staging of gastric cancer, especially when it is used in combination with other pre-operative examinations (62).

### Intra-Operative Staging

Intra-operative staging depends mainly on intra-operative exploration, and the development of laparoscopic technology has widely replaced traditional open laparotomy, with the associated advantages of minimally invasive approach besides the wide field of view. Laparoscopic exploration and analysis can help in the identification of laparoscopic metastases that are missed during imaging. Stell et al. (63) carried out a comparative study in 103 patients who underwent laparoscopic, ultrasound, and CT pre-operative staging, with resultant accuracy rates in detecting liver metastases of 99, 76, and 79%, respectively. Meanwhile, their comparative accuracy in identifying peritoneal metastasis was 94, 84, and 81%, respectively. Mirza and Galloway (64) reported that the overall sensitivity of laparoscopy, CT, and PET in the diagnosis of intra-abdominal metastases were 86, 81, and 78%, respectively. Meanwhile, their specificities in determining the N- and M-stage were (85, 82, and 79%) and (92, 79, and 89%), respectively. The accuracy of staging during laparoscopy was significantly higher than that of imaging examination.

In addition, laparoscopy has shown superiority over pre-operative imaging in determining the T and M stages of gastric cancer. Li et al. (65) retrospectively analyzed 1,067 patients with gastric cancer who underwent diagnostic laparoscopic. The results showed that the accuracy of laparoscopy in assessing whether gastric cancer invades adjacent organs was 98.3%, while its accuracy in detecting the presence of distant abdominal metastases was 98.1%. Therefore, a precise and individualized treatment plan cannot be separated from the staging of laparoscopy during the operation.

The correlation between pre-operative staging and prognosis in patients with gastric cancer has been examined in many reports; however, little is known about such correlation in the case of intra-operative staging. Koumori et al. (66) conducted an analysis to examine the value of intra-operative staging on survival outcomes in 915 individuals who had gastric resection for their gastric adenocarcinoma. There were 70.1, 1.6, 14.8, 12.1, 1.3, and 0.1% cases with stage I, IIA, IIB, III, IVA, and IVB, respectively. The authors noted that the hazard ratios of intra-operative staging for overall survival were 6.99, 2.23, 4.09, 6.06, and 14.92 for stages IIA, IIB, III, IVA, and IVB, respectively.

### Post-operative Staging

Although the rigorous staging before and during the surgical resection of gastric cancer is highly valued, the importance of staging after surgery cannot be denied. The International Alliance Against Cancer has always emphasized that post-operative staging (pTNM) is the gold standard for the staging of gastric cancer (67). The key to an accurate staging after surgery is the “completeness” of the surgeon's operation and the “integrity” of lymph node detection by the pathologist. Failure to remove a sufficient number of lymph nodes during surgery or failing to rigorously examine resected lymph nodes may negatively impact the accuracy of post-operative staging. In order to appropriately examine suspected lymph nodes, the pathologist should examine suspected lymph nodes for more than 3 times. Suspected metastatic lymph nodes that are difficult to identify by Hematoxylin and eosin (HE) staining can be further examined by immunohistochemical (IHC) staining.

Staging of lymph nodal metastasis is recognized as one of the critical factors affecting the prognosis of gastric cancer. The higher the stage of lymph node metastasis, the worse the prognosis. Since the fifth edition of TNM staging of gastric cancer, some major changes have been made to lymph node staging criteria. The new version no longer takes the distance between the anatomic site of the metastatic lymph node and the margin of the primary lesion (>3 cm) as a defining criterion for nodal metastasis (N2). When using the updated staging criteria for staging of lymph nodal metastasis, the total number of lymph nodes to be dissected is ≥15. Meanwhile, due to various reasons, the number of lymph nodes detected in some cases after surgery did not reach 15. Even in cases with more than 15 lymph nodes, the greater, the number of detected lymph nodes, the more positive lymph node results are. This, in turn, will have a significant impact on N staging. In this context, the bias associated with lymph nodal staging appeared, which cannot accurately predict the prognosis of patients with gastric cancer. Table 3 described the list of equipments used to evaluate pre-operative, inter-operative, and post-operative.

**Table 3:** List of equipments used to evaluate pre-operative, inter-operative, and post-operative.

**CBC analyzer machine**	**Infusion pump**
Electrocardiogram	Intra-aortic balloon pump
Pressure regulators	Pulse oximeter
Heart–lung bypass machine	Electrosurgical generators
Ventilator (or respirator)	Pulse lavage
Ultrasound machine	Visual display units

## Prognostic Evaluation

Analysis of large cases shows that the degree of tumor resection (R), the depth of tumor invasion (T), and lymph node metastasis (N) are the main critical factors that affect the prognosis of patients with gastric cancer. In addition, the correlation between prognosis and three important factors (metastasis rate of lymph nodes, number of negative lymph nodes, and free tumor cells in the abdominal cavity) has gradually attracted researchers' attention.

With the applications of the 7th edition of TNM staging, major adjustments have been applied to the T stage of the tumor. In the 6th edition, T2 stage refers to a tumor with a depth of invasion reaching the muscularis propria (T2a) or subserosa (T2b), which has been modified in the 7th edition as T2 (depth reaching muscularis propria) and T3 (depth reaching subserosa). In the 7th edition, T4 was divided into T4a and T4b, which was described when the gastric cancer is determined to reach the serosa or adjacent structures, respectively. The latter must be combined with organ resection to obtain R0 surgery. Park et al. (68) found that the disease-specific, 5-year survival rate to be significantly higher in patients with pT2a stage (based on the sixth edition of TNM staging classification) compared to pT2b gastric cancer (85.5 vs. 55.7%, *P* < 0.001). Also, the prognosis of patients with pT2a gastric cancer was significantly better than that of patients with pT2b or any pN stage (*P* < 0.001).

Deng et al. (69) analyzed the differences and the associated prognosis between T staging and lymph node staging in 395 gastric cancer patients who underwent D2 surgery. The results showed that in the TNM staging, there was an overlap between the survival curves of N2 and N3 patients (*P* > 0.05), the same was observed after the analysis of the survival curves of N2 and N3 patients by the lymph node staging of the Japanese Gastric Cancer Association (*P* > 0.05). Moreover, a paired statistical analysis of the number of lymph nodes in 308 patients with early-stage gastric cancer, who underwent radical gastric resection, found that the optimal cut-off value for metastatic lymph nodes should be considered: 0, 1–4, 5–8, and ≥9 nodes. Therefore, it was suggested that gastric cancer patients with 0, 1–4, 5–8, and >9 positive nodes may represent the four appropriate prognostic groups and should be adopted for the classification of the nodal stage in gastric cancer (70).

There is a clear and strong correlation between the ratio of metastatic to examined lymph nodes (N ratio or Nr). Maduekwe et al. (71) examined 257 patients with gastric cancer who underwent gastric resection and D1 lymphadenectomy during the period from 1995 to 2005. The authors determined the N ratio intervals as follows: Nr0: N ratio equals to 0 out of at least 15 examined nodes, Nr1 where the N ratio is between 0 and 0.3; Nr2 where the N ratio is between 0.3 and 0.7, and Nr3 where the N ratio is above 0.7.

Patients had a median number of resected lymph nodes of 14. The authors noted that the OS as stratified by the N status was significantly different in those with a resected number of lymph nodes of <15 compared to those with ≥15. However, when patients were stratified by N ratio intervals, the results did not reveal a significant difference in the two categories (<15 and ≥15 lymph nodes). Furthermore, the author conducted a multivariate analysis, and they found that the N ratio, not the N status, remained a significant independent prognostic factor.

Free abdominal tumor cells are also acknowledged as a critical factor in determining the prognosis of patients with gastric cancer; however, the clinical diagnosis of free tumor cells in the abdominal cavity lacks consensus standards and has low sensitivity.

## Clinical Trials for The Treatment of Gastric Cancer

Earlier, gastric cancer was being treated using minimally invasive methods such as laparoscopic surgery and endoscopic treatment. Because of the results of large clinical trials, surgery with extended lymphadenectomy could not be used as standard therapy for advanced gastric cancer. Therefore, few clinical trials are going for the treatment of gastric cancer including a Phase Ib/II, open-label, multi-center study designed to assess the tolerability, safety, tolerability, pharmacokinetics, and preliminary anti-tumor activity of immunotherapy-based treatment combinations in patients with gastric cancer (72).

Another study is carrying out to evaluate the safety and determine the best dose of PRS-343 immunotherapy for patients with advanced HER2-positive stomach cancers. PRS-343 is a protein that targets HER2 and an immune receptor (CD137). PRS-343 stimulates CD137-containing immune cells to group together and attach with HER2-containing tumor cells. This attachment may allow CD137 to stimulate the immune system to attack the HER2-positive cancer cells (9).

Different studies are also conducting to determine drug effects in the treatment of gastric cancer including Nivolumab in combination with other therapies, tucatinib combined with trastuzumab and modified CAPOX or FOLFOX7, APR-246 in combination with pembrolizumab and Oradoxel for advanced malignancies.

## AI-Based Gastric Cancer Treatment and Management

In gastric cancer, AI is mainly used for molecular bio-information analysis, chronic atrophic gastritis, early gastric cancer, endoscopic detection for *H. pylori* infection, pathology recognition, and invasion depth. AI may also be used to establish predictive models for evaluating response to drug treatments, lymph node metastasis, and prognosis (73).

Luo et al. have constructed the Gastrointestinal Artificial Intelligence Diagnostic System that can automatically detect upper gastrointestinal cancer in real-time. An artificial intelligence (AI)-based endoscopic diagnosis system has also been developed for the early identification of gastric cancer. Another study presented the convolutional neural network (CNN) model to automatically detect tumor-infiltrating lymphocytes (TILs) on histopathological whole side imaging (WSI) with an acceptable accuracy of 96.88% (74).

## Challenges of Drug Resistance

The main reason for treatment failure for gastric cancer is the development of multidrug resistance (MDR). Recently, research on MDR gastric cancer has revealed that, in addition to the classical ATP-binding cassette transporters including P-glycoprotein (P-gp) and MDR-associated protein (MRP)1, various other molecules might mediate the drug resistance of gastric cancer. The absence of MRP1 and P-gp expression in some gastric cancer cases also shows that there might be other mechanisms responsible for human gastric cancer MDR.

The keys to overcoming drug resistance problems in gastric cancer include a collection of appropriate clinical tissue samples before and after chemotherapy and at relapse, correlation with high-quality clinical data, chemosensitivity tests, and determination of the main drug resistance-associated molecules involved (75).

## Summary of Trials and its Major Changes

In a systematic review of 388 trials with surgery, most trials used a superiority design (84.8%) and were designed to detect a large treatment effect. Only 31.7% used major clinical events as the primary outcome. In most trials 78.1% did not control for surgeon experience, only 4.4% evaluate the quality of the intervention. In most trials, 54.4% had some concern of bias and 23.5% had a high risk of bias. While other trials 54.6% reported a neutral result; reporting bias was identified in 51.7%. Multiplicity was detected in 45.1%, and only 20.0% adjusted for multiple comparisons (76). In another trial, 117 individuals were randomized between control (surgery alone), intra-arterial chemotherapy and systemic chemotherapy. Forty percent of patients relapsed in the control group and 33% in the chemotherapy group (NS). For male patients, the difference in disease-free survival was significant, though not in women (77).

## Summary

The most preferred treatment option for patients with advanced gastric cancer is surgical resection. Meanwhile, in certain cases, where the surgical approach cannot be applied, the main goal of comprehensive treatment is to prolong survival and to improve the quality of life. Despite the fact that the number of clinical studies investigating other treatment modalities (neoadjuvant chemotherapy, targeted therapy, and immunotherapy) is scarce, the beneficial outcomes of the neoadjuvant chemotherapeutic approach cannot be ignored. The advent in novel chemotherapeutic drugs, targeted therapies, and recent advances in tumor molecular biology research will provide new opportunities for the comprehensive staging and treatment of advanced gastric cancer. This review highlights different treatment approaches used for gastric cancer treatment. However, there are still some limitations, like this review did not contain enough literature regarding clinical trials of different drugs used in the treatment of gastric cancer. Based on the observations in the literature, future research and novel advancements will make it possible to improve the treatment approaches of advanced gastric outcomes, with the subsequent substantial impact on patients' survival outcomes.

## Author Contributions

Z-dC and P-fZ wrote the manuscript. H-qX and BW collected the data. LC and YT analyzed the data. All authors contributed to the article and approved the submitted version.

## Funding

This work was supported by grants from the National Nature Science Foundation of China (Nos. 81972790, 81672319, 81602507, 81773135, and 81572465).

## Conflict of Interest

The authors declare that the research was conducted in the absence of any commercial or financial relationships that could be construed as a potential conflict of interest.

## Publisher's Note

All claims expressed in this article are solely those of the authors and do not necessarily represent those of their affiliated organizations, or those of the publisher, the editors and the reviewers. Any product that may be evaluated in this article, or claim that may be made by its manufacturer, is not guaranteed or endorsed by the publisher.
